# Assessment of the diagnostic accuracy of Vibrasense compared to a biothesiometer and nerve conduction study for screening diabetic peripheral neuropathy

**DOI:** 10.1186/s13047-023-00667-3

**Published:** 2023-09-28

**Authors:** Srihari Sharma K. N, Anil Kumar H

**Affiliations:** 1 College of Physiotherapy, Dayananda Sagar University, Shavige Malleshwara Hills, 1st Stage, Kumaraswamy Layout, Bangalore, Karnataka India 560111; 2 Department of Medicine, Dr Chandramma Dayananda Sagar Institute of Medical Education and Research (CDSIMER), Kanakapura, Karnataka India

**Keywords:** Diabetic peripheral neuropathy, Biothesiometer, Vibration perception threshold, Quantitative sensory testing, Type 2 diabetes mellitus

## Abstract

**Aims:**

Peripheral neuropathy is a common microvascular complication in diabetes and a risk factor for the development of diabetic foot ulcers and amputations. Vibrasense (Ayati Devices) is a handheld, battery-operated, rapid screening device for diabetic peripheral neuropathy (DPN) that works by quantifying vibration perception threshold (VPT). In this study, we compared Vibrasense against a biothesiometer and nerve conduction study for screening DPN.

**Methods:**

A total of 562 subjects with type 2 diabetes mellitus underwent neuropathy assessments including clinical examination, 10-g monofilament test, VPT evaluation with Vibrasense and a standard biothesiometer. Those with an average VPT ≥ 15 V with Vibrasense were noted to have DPN. A subset of these patients (*N* = 61) underwent nerve conduction study (NCS). Diagnostic accuracy of Vibrasense was compared against a standard biothesiometer and abnormal NCS.

**Results:**

Average VPTs measured with Vibrasense had a strong positive correlation with standard biothesiometer values (Spearman’s correlation 0.891, *P* < 0.001). Vibrasense showed sensitivity and specificity of 87.89% and 86.81% compared to biothesiometer, and 82.14% and 78.79% compared to NCS, respectively.

**Conclusions:**

Vibrasense demonstrated good diagnostic accuracy for detecting peripheral neuropathy in type 2 diabetes and can be an effective screening device in routine clinical settings.

**Trial registration:**

Clinical trials registry of India (CTRI/2022/11/047002). Registered 3 November 2022. https://ctri.nic.in/Clinicaltrials/pmaindet2.php?trialid=76167.

**Supplementary Information:**

The online version contains supplementary material available at 10.1186/s13047-023-00667-3.

## Introduction

Diabetic peripheral neuropathy (DPN) is a common microvascular complication that affects nearly 50% of people with diabetes within their lifetime [1]. It most frequently affects the peripheral regions including the lower limbs and hands in a “stocking-glove” distribution [2]. DPN has been implicated as an important etiopathological factor leading to diabetic foot ulcers and resultant lower-extremity amputations [3, 4].

Early detection of DPN and implementation of appropriate preventive measures are important elements in the management of individuals with diabetes. International guidelines from various professional associations have recommended periodic screening, at least annually, for DPN [4–6]. Some of the commonly suggested methods for screening include history-taking for neuropathy symptoms, evaluation of temperature and pinprick sensations (for small-fiber function), vibration perception testing (for large-fiber function), and the use of a 10-g monofilament (for assessing protective sensation) [4, 5].

The standard 128-Hz tuning fork is the traditional and easy method to detect the presence of vibration perception [4, 5]. However, the 128-Hz tuning fork does not provide quantitative information about the degree of loss of vibration sensation. Quantitative testing of vibration sensation is important as impaired vibration perception can also be predictive of the risk of foot ulceration [7–9]. The Rydel-Seiffer graduated tuning fork can evaluate vibration perception threshold (VPT) on a scale of 0–8 and has been standardized for quantitative sensory testing (QST) [10]. Several electromechanical devices have also been developed for quantitative sensory testing of vibration perception. These devices known generically as digital biothesiometers have been reported to have several advantages including consistent delivery of vibration stimulus, greater range of vibrational intensities, and strong reliability and accuracy [11, 12]. Previous research findings have demonstrated that VPT measured with biothesiometers exhibits favorable diagnostic accuracy in the identification of DPN when compared to clinician diagnosis, neuropathy symptom scores, and abnormal nerve conduction [13–16].

According to the latest estimates by the International Diabetic Federation (IDF), India had approximately 74 million adults with diabetes in 2021, which is projected to increase to 92.9 million by 2030 [17]. To prevent the burden of foot ulcers in India, it is necessary that physicians implement regular DPN screening in their practices [18, 19]. Published studies from India indicate that VPT evaluation is being used for the detection of DPN in secondary- and tertiary-care centers [20–27]. However, these devices have not gained widespread usage in primary care practice. Some of the factors that may be limiting their use in routine clinical settings include their size, the requirement for a dedicated space and uninterrupted power supply, and the time needed for the examination [28, 29].

Vibrasense (Ayati Devices) is a compact, handheld screening device that measures VPT to screen for DPN. It presents several benefits over the other commonly available biothesiometry devices, including its light-weightedness, rechargeability, and smart functionalities for usage with a mobile device. There have been no published studies comparing this device against the commonly used biothesiometers. In this prospective study, we compared the quantitative VPT measured with Vibrasense to a standard office biothesiometer. Additionally, we evaluated the diagnostic accuracy of Vibrasense for DPN diagnosis by comparing it to the comparator biothesiometer and abnormal nerve conduction study (NCS).

## Subjects, materials and methods

### Study design and participants

The study was conducted at Dr.Chandramma Dayananda Sagar Institute of Medical Education and Research (CDSIMER) located at Kanakapura, Karnataka, India, between November 2022 to December 2022. The study was approved by the Institutional Ethics Committee and complied with the Declaration of Helsinki. The trial was registered in the clinical trials registry of India (CTRI/2022/11/047002).

Individuals with diabetes visiting the Medicine OPD and participating in the diabetic camps conducted by the hospital were screened based on the study selection criteria. After explaining the study procedures, consent was obtained from all patients prior to enrollment in the study. The study included adult patients (> 18 years) with type 2 diabetes mellitus (T2DM) of any duration confirmed by medical records. The study excluded individuals with active foot ulcers or visible signs of recently healed foot ulcers, a history of any lower limb amputation, or a prior diagnosis of peripheral neuropathy of any cause.

The study assessments took place in a specified room within the Medicine department and in dedicated spaces arranged for this purpose at diabetes camps. AKH, with 17 years of clinical experience in Internal Medicine, managed the screening and selection of patients for the study, while SSKN, with 15 years of clinical experience in physical therapy and training in biothesiometry, was responsible for recording the study measures, including the monofilament tests and vibration perception tests. NCSs were performed by CPA, a qualified electrophysiologist with more than 20 years of experience.

Demographic details, history of diabetes, details of glycemic control, history of other comorbidities like hypertension, and presence of any neuropathy symptoms were recorded. Height and weight were assessed with the subject barefoot, utilizing a wall-mounted wooden stadiometer and an electronic scale, respectively. Body Mass Index (BMI) was derived by dividing body weight in kilograms by the square of height in meters (kg/m^2^). Subsequently, the feet were inspected to identify skin irregularities including excessively dry skin, calluses, fissures, as well as deformities such as claw or hammer toes, hallux valgus, joint subluxation, prominent metatarsal heads, and medial convexity, among others. This was followed by neuropathy evaluation that was consistently performed on the right foot. All patients were subjected to monofilament test and vibration perception threshold (VPT) testing with Vibrasense and a comparator office biothesiometer. As per the study protocol, a randomly selected subset of the participants, comprising approximately 10% of the total study population (*N* = 61), was subjected to a NCS. Those detected with DPN in any of the diagnostic tests were provided counseling about neuropathy, its possible consequences, and the importance of diet, exercise, foot care, and compliance with medications.

### Study procedures

#### Semmes–Weinstein 10-g monofilament examination

Protective sensation was evaluated using a 10-g Semmes–Weinstein monofilament (Diabetik Foot Care India Pvt Ltd, India). The monofilament was applied to the palm first to make the subject familiar with the sensation. With the subject’s eye closed, the monofilament was applied perpendicular to the skin surface with enough force to make it bend or buckle. Out of the nine sites recommended in the plantar surface of the foot, we tested six points, which included four sites in the forefoot (great toe, first-, third-, and fifth-metatarsal heads), one site in the midfoot (medial side of the midfoot) and one site in the hindfoot (heel) [18, 30]. The subject was instructed to say “yes” when the monofilament was sensed on the foot. The inability to sense the monofilament at one or more points was defined as abnormal [18, 30].

#### Vibration perception thresholds

VPTs were assessed using Vibrasense (Ayati Devices Private Ltd, India) and a standard Biothesiometer (Biothezi VPT from Kody Medical Electronics Private Ltd, India). Both devices produce vibration amplitudes from 0.026–25 microns expressed as vibration units that range from 1—50 V, where a higher unit of vibration stimuli indicates a greater sensory loss. Following the protocol recommended by both manufacturers, and consistent with other published Indian studies, VPT measurements were taken at six specific points on the plantar surface of the foot: the great toe, the first-, third-, and fifth-metatarsal heads, the medial side of the midfoot, and the heel [22–24, 27]. The test was explained to the patient and the vibration probe was applied to the hand of the subject to familiarize them with the expected vibratory sensation. After this, with the subject’s eyes closed, vibration perception was tested at the six points of the foot. The subject was asked to say “yes” when a buzzing sensation was felt on the foot. The vibration stimulus was started at 1 V and was gradually increased until the subject reported feeling the sensation. The lowest vibrational intensity sensed at each point was taken as the VPT. The average VPT of all six points tested in the foot was then recorded for each patient.

#### Nerve conduction study

Sixty-one subjects underwent sural and peroneal motor nerve conduction study on the right leg with a 4-channel electromyograph (EMG-Octopus, model CMEMG-01). Distal latencies, amplitude of action potentials, and conduction velocities were recorded in the sural and peroneal nerves in the right leg. DPN was diagnosed when there were one or more abnormal nerve conduction parameters in the sural sensory nerve and the peroneal motor nerve using standard electrophysiological criteria [31].

### Statistical analysis

Data are presented as mean (n), proportions (%), and distribution as mean with standard deviations. Mann-Whitney U test was performed to evaluate the differences in the VPTs measured by either device. Spearman correlation analysis was used to determine the relationship between the average VPT values measured by Vibrasense and the comparator Biothesiometer. A *p*-value < 0.05 was considered statistically significant. DPN diagnosis was made when the average VPT was at or above 15 V, as defined by the manufacturers of both devices. The sensitivity and specificity of Vibrasense for DPN diagnosis were calculated with Biothesiometer as the reference standard (*N* = 562) and in the subset analysis with abnormal NCS as the reference standard (*N* = 61). Receiver operating characteristic (ROC) curve was plotted to compare the diagnostic performance of Vibrasense against NCS-diagnosed DPN and the area under the curve (AUC) was determined. The resulting area under the curve (AUC) helps interpret the discriminatory capability of a test and was categorized as follows: Fail (0.50—0.59), Poor (0.60—0.69), Fair (0.70—0.79), Good (0.80—0.89), and Excellent (≥ 0.90) [32]. Some guidelines suggest that a VPT threshold of 25 V strongly predicts future foot ulceration risk [3, 33]. Therefore, besides the manufacturer’s recommended diagnostic threshold of 15 V, we also conducted an exploratory analysis examining the diagnostic efficacy of Vibrasense against abnormal NCS using a 25 V cut-off. Statistical analyses were performed with IBM SPSS Statistics, Version 25.

## Results

A total of 562 subjects with type 2 diabetes mellitus were enrolled in the study. The baseline characteristics of the study subjects are given in Table 1. The mean age of the participants was 56.42 ± 12.57 years with almost equal gender distribution (M:F, 1:1.05). The mean (± SD) duration of T2DM was 5.93 (± 4.75) years, and ranged from 1 month to 35 years. One or more symptoms of neuropathy was present in approximately 64% subjects including burning, aching pain or tenderness in the legs or feet (43.1%), prickling sensations (39.9%), numbness (33.1%), and unsteadiness in walking (13%). Skin abnormalities in the foot such as dryness, callus, fissures and other changes were noted in 56% of the subjects.

**Table 1:** General characteristics of the study group

Characteristic	Value (*N* = 562)
Age (years, Mean ± SD)	56.42 ± 12.57
Gender (M:F)	274: 288
BMI (kg/m^2^, Mean ± SD)	26.30 ± 5.59
Duration of Diabetes (years, Mean ± SD)	5.93 ± 4.75
Last Random Blood Sugar (RBS) (mg/dL, Mean ± SD)	246.97 ± 94.84
History of hypertension (n, %)	176 (31.3%)
Neuropathy symptoms (n, %)	363 (64.6%)
• Unsteadiness in walking	73 (13.0%)
• Numbness	186 (33.1%)
• Pain or tenderness in legs or feet	242 (43.1%)
• Prickling sensations	224 (39.9%)
Foot skin appearance (n, %)	
• Abnormal (dryness, callus, fissures, etc.)	315 (56%)

### Comparison with biothesiometer

VPT values recorded with Vibrasense and the standard biothesiometer are compared in Table 2. The mean VPT values measured with Vibrasense at the six points matched closely with the values measured with the comparator Biothesiometer.

**Table 2:** Comparison of mean VPT values with Vibrasense and biothesiometer (*N* = 562)

**Parameter**	**Vibrasense VPT** Mean ± SD	**Biothesiometer VPT** Mean ± SD	***P* value**^a^
VPT at Point 1	21.61 ± 12.01	21.56 ± 12.69	0.827 (NS)
VPT at Point 2	21.18 ± 12.33	21.25 ± 12.86	0.997 (NS)
VPT at Point 3	20.97 ± 12.19	21.70 ± 13.03	0.458 (NS)
VPT at Point 4	21.37 ± 12.30	21.81 ± 13.13	0.698 (NS)
VPT at Point 5	21.94 ± 12.64	21.93 ± 13.40	0.821 (NS)
VPT at Point 6	21.83 ± 12.43	22.00 ± 13 34	0.853 (NS)
Average VPT of Foot	23.02 ± 13.33	22.96 ± 13.70	0.867 (NS)

*NS* Not significant

^a^Mann-Whitney U-test

**Fig. 1:**
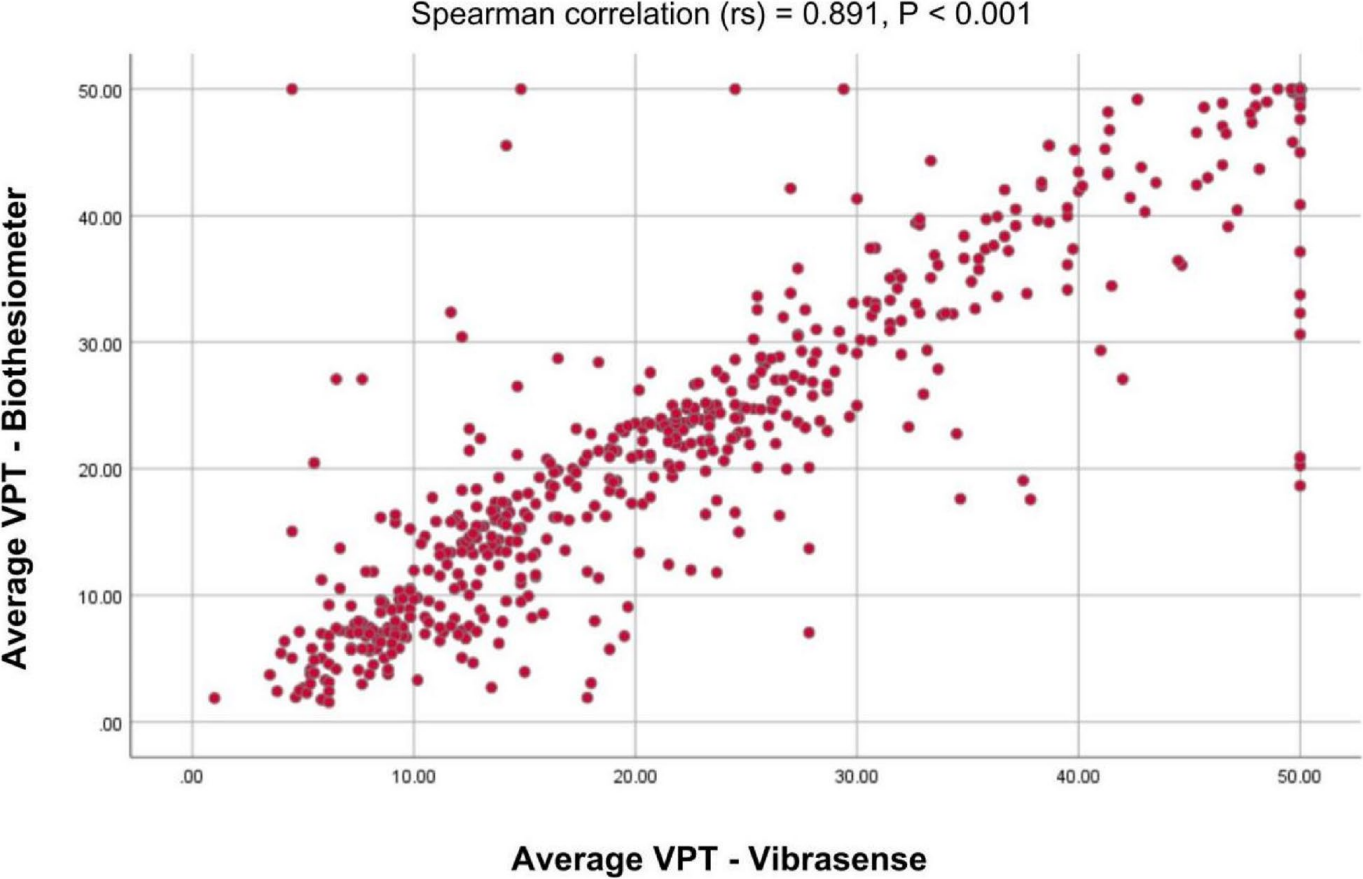
Correlation of Vibrasense average VPTs vs biothesiometer average VPTs

Average VPT of the foot recorded with Vibrasense showed a very strong positive correlation (Spearman’s correlation *r*_s_ = 0.891, *P* < 0.001) with average VPT values recorded with Biothesiometer (Fig. 1).

DPN as defined by VPT ≥ 15 was noted in 358 (63.7%) subjects with Vibrasense and 380 (67.6%) subjects with the comparator biothesiometer. When the standard biothesiometer was considered as the reference test for DPN, Vibrasense had a sensitivity of 87.89% and specificity of 86.81% (Table 3).

**Table 3:** Sensitivity and specificity of Vibrasense based on biothesiometer as the reference standard (*N* = 562)

	**Biothesiometer diagnosis**		
**Vibrasense diagnosis**	Neuropathy	No neuropathy	Total
Neuropathy	334	24	358
No Neuropathy	46	158	204
Total	380	182	562
• Sensitivity: 87.89%			
• Specificity: 86.81%			
• Positive Predictive Value (PPV): 93.30%			
• Negative Predictive Value (NPV): 77.45%			

### Comparison with NCS

Among the study participants, 61 underwent the NCS test for DPN assessment. DPN diagnosis was made by NCS in 28 (45.9%) of these individuals. When abnormal NCS was taken as the reference test for DPN, Vibrasense had a sensitivity of 82.14% and specificity of 78.79% (Table 4). The ROC curve analysis revealed that Vibrasense had an AUC of 0.839 (95% CI: 0.730 to 0.948, *P* < 0.001), which indicates good discriminatory power for DPN diagnosis (See Supplementary Fig. 1, Additional file 1) [32]. The exploratory analysis with VPT ≥ 25 V cut-off demonstrated a lower sensitivity of 57.14%, but a higher specificity of 90.90%. The 10-g monofilament test showed a sensitivity of only 35.7% but had a high specificity of nearly 91% versus abnormal NCS.

**Table 4:** Diagnostic accuracy of Vibrasense and monofilament tests compared to NCS

**Tests comparison**	**NCS diagnosis**	**Normal (*N* = 33)**		**Abnormal (*N* = 28)**	
	**Test diagnosis**	**Positive**	**Negative**	**Positive**	**Negative**
	Vibrasense	7	26	23	5
	10-g monofilament	3	30	10	18
**Diagnostic accuracy**	**Parameter**	**Sensitivity**	**Specificity**	**PPV**	**NPV**
	Vibrasense	82.14%	78.79%	76.67%	83.87%
	10-g monofilament	35.7%	90.90%	76.92%	62.5%

## Discussion

In the present study, it was found that the portable neuropathy screening device Vibrasense yielded VPT measurements that closely corresponded to those obtained using the standard office biothesiometer. Vibrasense exhibited good sensitivity and specificity for DPN diagnosis compared to the comparator biothesiometer. These findings support the use of Vibrasense as a suitable alternative to the standard office biothesiometer for the screening DPN in people with type 2 diabetes. In comparison to the conventional office biothesiometer, Vibrasense offers several benefits such as its portable and lightweight nature, battery-operated functionality, and smart features that facilitate report generation via bluetooth connectivity to a mobile device. These attributes render Vibrasense an attractive point-of-care device for screening DPN. Moreover, the capacity to assess more than 70 patients on a single charge makes it particularly suitable for deployment in diabetic camps and settings with unstable power supplies.

The Vibrasense mobile app creates a report with a gradient of colors reflecting vibration perception levels: transitioning from blue for normal (< 15 V), through green for mild impairment (15–20 V) and yellow for moderate impairment (20–25 V), to red indicating severe impairment of vibration perception (> 25 V). By providing a clear visual representation of areas of impaired sensation, healthcare providers can enhance patient understanding and empower them to take a more active role in their care. Healthcare professionals can use it as a visual aid to educate patients about the importance of optimal diabetes management, appropriate foot care, and regular monitoring of DPN.

The obtained sensitivity of 82.14% and specificity of 78.79% for Vibrasense against the abnormal NCS test are clinically acceptable, particularly considering the context of Vibrasense as a screening tool. Our study findings are consistent with the previous VPT-based diagnostic studies that have reported sensitivities ranging from 66.5% to 86% and specificities ranging from 52.9% to 86.6% against NCS [13, 15, 16, 22, 26]. The cost of Vibrasense-based DPN screening is approximately one-tenth the cost of NCS in our clinical setting. The low cost of the test with good diagnostic accuracy will enable healthcare providers to screen a larger number of patients more efficiently and cost-effectively.

Our findings indicate that a VPT diagnostic threshold of 15 V when using Vibrasense demonstrated effective discriminatory ability in detecting DPN, as reflected by an AUC of 0.839. Other studies from India that have used VPT ≥ 15 V in the plantar foot as the criteria for DPN diagnosis have also reported acceptable sensitivities and specificities versus abnormal NCS [16, 22]. A higher VPT cut-off of 25 V has been used in some studies based on its ability to predict foot ulceration [3, 9, 34]. Prior research has reported that the higher VPT thresholds for diagnosis result in better specificity but reduce sensitivity [15, 35, 36]. Our exploratory analysis also confirmed this finding with a lower sensitivity of 57.14% with the diagnostic cut-off of 25 V, compared to 82.14% with 15 V. Similar findings of lower sensitivity with 25 V have been reported by another group from India [16]. A study conducted among Chinese subjects with type 2 diabetes reported that the optimal VPT cut-off was > 14.9 V, with a sensitivity of 66.5% and specificity of 77% against abnormal nerve conduction, compared to a cut-off of 25 V, which had a lower sensitivity of 48.4% and specificity of 92.5% [15]. The findings of the present study suggest that a VPT cut-off of 15 V is suitable for routine screening of DPN with Vibrasense. A higher cut-off of 25 V may result in missing some early cases of DPN with milder degrees of impairment in vibration perception.

Guidelines have recommended the 10-g monofilament test to detect loss of protective sensation (LOPS) because of its favorable evidence in predicting the risk of foot ulceration [3, 7, 37, 38]. The monofilament test is a commonly used and inexpensive method for screening DPN. In the current study, the 10-g monofilament demonstrated a lower sensitivity of 35.7% and a higher specificity of ~ 91% for the detection of DPN against abnormal NCS. This is in line with other studies that have reported low sensitivities and high specificities for the monofilament test against NCS [16, 39]. A systematic review of 19 trials reported a pooled sensitivity of 53% and specificity of 88% for monofilament-based detection of DPN [40]. Neuropathy assessment using 20 different Semmes–Weinstein monofilaments that exert forces from 0.008 to 300 g (labeled on a logarithmic scale between 1.65 to 6.65) is a standardized method of performing Quantitative sensory testing (QST) [41]. However, the 5.07/10 g monofilament is the only filament used in routine clinical practice [5, 7]. The 10-g monofilament can detect those with the highest risk of foot complications [38]. It is an effective method to identify those with advanced neuropathy [42, 43]. However, it can miss some early cases that can be detected in VPT evaluation [44, 45]. This may explain the lower sensitivity of monofilament compared to VPT testing in this study. The International Working Group on the Diabetic Foot (IWGDF) guidelines recommends adjunctive screening of vibration perception with a tuning fork or biothesiometer when a monofilament test does not show any loss of protective sensations [46]. In line with this recommendation, our study suggests that the inclusion of VPT testing can identify additional cases of DPN that can be missed by the 10-g monofilament.

Another notable advantage of VPT testing is its ability to provide quantified vibration perception values, which can be utilized to actively monitor individuals at risk and implement targeted educational interventions to improve neuropathy-related measures [47–49]. The software provided with the Vibrasense device includes a long-term graph feature that allows for the plotting of average VPT progression over time. This functionality can serve as a valuable tool for physicians and patients in tracking changes in areas exhibiting diminished vibration sensation over an extended period.

In our study, we observed that the VPT-based prevalence of DPN was 63.7% with Vibrasense and 67.6% with the comparator biothesiometer. Previous hospital-based studies from India based on VPT assessments in individuals with type 2 diabetes mellitus have documented prevalence rates of DPN ranging from 18.75% to 38% [16, 20, 50, 51]. However, it is important to note that most of these studies used a VPT cut-off of 25 V for diagnosis, while our study used VPT 15 V for diagnosis, which may have resulted in the higher reported prevalence in our study. One of these studies also documented that the estimated prevalence of DPN was 18.75% with VPT 25 V but increased to 56.25% when VPT 15 V was used as the diagnostic cut-off [16]. In the present study, NCS-diagnosed DPN was seen in 45.9% of subjects tested. This is within the range (29-71%) of prevalence reported by previous electrophysiological studies in type 2 diabetes subjects from India [16, 22, 52, 53].

One of the limitations of this study is that it included only people with type 2 diabetes, and the diagnostic accuracy of the device in type 1 diabetes needs to be investigated in future studies. We used impaired VPT which evaluates only large fiber dysfunction, while small fiber neuropathy was not tested which may be considered a limitation when making estimates of DPN prevalence. The participants in the study were predominantly from rural areas with primarily low socioeconomic status, exhibiting inadequate glycemic control (as indicated by the mean RBS of 246 mg/dL); thus, caution should be exercised when generalizing these findings to urban populations. In an urban population-based study conducted in Chennai among individuals with type 2 diabetes mellitus, utilizing a VPT threshold of ≥ 20 for diagnosis, a raw prevalence rate of 26.1% for DPN was reported [54]. Another population-based study conducted Chennai that longitudinally followed urban type 2 diabetes patients documented a four-year DPN incidence of 28% using VPT ≥ 20 as the cutoff point [55]. Our study population comprised diabetic patients attending outpatient clinics and camps, who are more likely to exhibit complications. Consequently, it is plausible that the observed prevalence in our study is an overestimate of the actual population prevalence of DPN in India. We performed VPT testing at 6 points in the plantar foot as per the protocol recommended by the manufacturers and followed by many centers across India as evidenced from published studies [22–24, 27]. The other established protocol is testing a single site on the hallux [9, 20, 21, 51]. Future research can validate the diagnostic accuracy of Vibrasense using the single-site protocol, which can offer substantial time efficiency for medical practitioners.

## Conclusions

In conclusion, the Vibrasense device exhibited favorable diagnostic accuracy in detecting DPN when compared to the standard office biothesiometer and abnormal nerve conduction study. Our study findings suggest that a VPT diagnostic threshold of 15 V using Vibrasense had a strong discriminatory ability for DPN detection. These results support the utility of Vibrasense as a valuable tool in routine clinical practice for screening diabetic peripheral neuropathy, presenting it as a suitable alternative to the standard office biothesiometer. The device’s portability and rechargeability can offer significant benefits in community settings and during diabetic camps.

### Supplementary Information

**Additional file 1.** ROC curve analysis of diagnostic performance of Vibrasense  against NCS-diagnosed DPN. ROC curve analysis was used to evaluate the diagnostic performance of Vibrasense against NCS-diagnosed DPN. The green diagonal line represents a classifier that makes random predictions. The blue line illustrates the diagnostic performance at different VPT levels of Vibrasense. The closer this curve is to the upper left corner of the plot, the better the performance.

## Data Availability

The datasets during and/or analysed during the current study available from the corresponding author on reasonable request.

## References

[1] Hicks CW, Selvin E (2019). Epidemiology of peripheral neuropathy and lower extremity disease in diabetes. Curr Diab Rep.

[2] Feldman EL, Callaghan BC, Pop-Busui R, Zochodne DW, Wright DE, Bennett DL, Bril V, Russell JW, Viswanathan V (2019). Diabetic neuropathy. Nat Rev Dis Primers.

[3] Boulton AJ, Armstrong DG, Albert SF, Frykberg RG, Hellman R, Kirkman MS, Lavery LA, Lemaster JW, Mills JL, Mueller MJ, Sheehan P, Wukich DK, American Diabetes Association; American Association of Clinical Endocrinologists (2008). Comprehensive foot examination and risk assessment: a report of the task force of the foot care interest group of the American Diabetes Association, with endorsement by the American Association of Clinical Endocrinologists. Diabetes Care.

[4] Schaper NC, van Netten JJ, Apelqvist J, Bus SA, Fitridge R, Game F, Monteiro-Soares M, Senneville E. IWGDF Editorial Board. Practical guidelines on the prevention and management of diabetes-related foot disease (IWGDF 2023 update). Diabetes Metab Res Rev. 2023:e3657. 10.1002/dmrr.3657. 10.1002/dmrr.3657 37243927

[5] ElSayed NA, Aleppo G, Aroda VR, Bannuru RR, Brown FM, Bruemmer D, Collins BS, Gibbons CH, Giurini JM, Hilliard ME, Isaacs D, Johnson EL, Kahan S, Khunti K, Leon J, Lyons SK, Perry ML, Prahalad P, Pratley RE, Seley JJ, Stanton RC, Sun JK, Gabbay RA, on behalf of the American Diabetes Association (2023). 12. Retinopathy, neuropathy, and foot care: standards of care in diabetes-2023. Diabetes Care.

[6] Cosentino F, Grant PJ, Aboyans V, Bailey CJ, Ceriello A, Delgado V, Federici M, Filippatos G, Grobbee DE, Hansen TB, Huikuri HV, Johansson I, Jüni P, Lettino M, Marx N, Mellbin LG, Östgren CJ, Rocca B, Roffi M, Sattar N, Seferović PM, Sousa-Uva M, Valensi P, Wheeler DC, ESC Scientific Document Group (2020). 2019 ESC Guidelines on diabetes, pre-diabetes, and cardiovascular diseases developed in collaboration with the EASD. Eur Heart J..

[7] Bus SA, Lavery LA, Monteiro-Soares M, Rasmussen A, Raspovic A, Sacco ICN, van Netten JJ, International Working Group on the Diabetic Foot (2020). Guidelines on the prevention of foot ulcers in persons with diabetes (IWGDF 2019 update). Diabetes Metab Res Rev.

[8] Armstrong DG, Boulton AJM, Bus SA (2017). Diabetic Foot Ulcers and Their Recurrence. N Engl J Med.

[9] Young MJ, Breddy JL, Veves A, Boulton AJ (1994). The prediction of diabetic neuropathic foot ulceration using vibration perception thresholds A prospective study. Diabetes Care.

[10] Rolke R, Baron R, Maier C, Tölle TR, Treede DR, Beyer A, Binder A, Birbaumer N, Birklein F, Bötefür IC, Braune S, Flor H, Huge V, Klug R, Landwehrmeyer GB, Magerl W, Maihöfner C, Rolko C, Schaub C, Scherens A, Sprenger T, Valet M, Wasserka B (2006). Quantitative sensory testing in the German Research Network on Neuropathic Pain (DFNS): standardized protocol and reference values. Pain.

[11] Bril V, Kojic J, Ngo M, Clark K (1997). Comparison of a neurothesiometer and vibration in measuring vibration perception thresholds and relationship to nerve conduction studies. Diabetes Care.

[12] Temlett JA (2009). An assessment of vibration threshold using a biothesiometer compared to a C128-Hz tuning fork. J Clin Neurosci.

[13] Martin CL, Waberski BH, Pop-Busui R, Cleary PA, Catton S, Albers JW (2010). Vibration perception threshold as a measure of distal symmetrical peripheral neuropathy in type 1 diabetes: results from the DCCT/EDIC study. Diabetes Care.

[14] Santos TRM, Melo JV, Leite NC, Salles GF, Cardoso CRL (2018). Usefulness of the vibration perception thresholds measurement as a diagnostic method for diabetic peripheral neuropathy: results from the Rio de Janeiro type 2 diabetes cohort study. J Diabetes Complications.

[15] Liu M, Gao Y, Chen DW, Lin S, Wang C, Chen LH, Ran XW (2021). Quantitative vibration perception threshold in assessing diabetic polyneuropathy: should the cut-off value be adjusted for Chinese individuals with type 2 diabetes?. J Diabetes Investig.

[16] Ramanathan S, Thomas R, Chanu AR, Naik D, Jebasingh F, Sivadasan A (2021). Standard clinical screening tests, sural radial amplitude ratio and F wave latency compared to conventional nerve conduction studies in the assessment of sensorimotor polyneuropathy in patients with type 2 diabetes mellitus. Indian J Endocr Metab.

[17] International Diabetic Federation (IDF). India Diabetes report 2000 - 2045. Accessed 5 Jan 2023. https://www.diabetesatlas.org/data/en/country/93/in.html.

[18] Mishra SC, Chhatbar KC, Kashikar A, Mehndiratta A (2017). Diabetic foot. BMJ.

[19] Mehndiratta A, Mishra SC, Bhandarkar P (2020). Increasing identification of foot at risk of complications in patients with diabetes: a quality improvement project in an urban primary health centre in India. BMJ Open Qual.

[20] Jayaprakash P, Bhansali A, Bhansali S, Dutta P, Anantharaman R, Shanmugasundar G, Ravikiran M (2011). Validation of bedside methods in evaluation of diabetic peripheral neuropathy. Indian J Med Res.

[21] Gill HK, Yadav SB, Ramesh V, Bhatia E (2014). A prospective study of prevalence and association of peripheral neuropathy in Indian patients with newly diagnosed type 2 diabetes mellitus. J Postgrad Med.

[22] Mythili A, Kumar KD, Subrahmanyam KA, Venkateswarlu K, Butchi RG (2010). A Comparative study of examination scores and quantitative sensory testing in diagnosis of diabetic polyneuropathy. Int J Diabetes Dev Ctries.

[23] Kaur J, Batra AP (2016). Vibration perception threshold as a measure of distal symmetrical neuropathy in type 2 diabetes. Int J Contemp Med Res.

[24] Dash S, Thakur AK (2017). Perception of vibration threshold is a marker of diabetic neuropathy. Natl J Physiol Pharm Pharmacol.

[25] Aruna B, Haragopal R (2017). Role of Biothesiometry in the diagnosis of diabetic neuropathy. Indian J Clin Anat Physiol.

[26] Bharathi C, Roopakala MS, Shivaprasad C, Acharya PT (2018). Diagnostic value of vibration perception threshold in diabetic peripheral neuropathy. Int J Physiol.

[27] Devi M, Singh S, Dhanawat M, Gupta K, Gupta S, Agarwal BK, Verma I, Nain P (2022). Assessment of peripheral neuropathy pain by biothesiometer in diabetes mellitus patients. J Young Pharm.

[28] Todd O’Brien (2019). Evidence-based assessment of pediatric diabetic peripheral neuropathy. Curre Res Diabetes Obes J.

[29] Lee EW, Kang SY, Jang EC, Jin WJ, Seo KM (1995). Quantitative sensory test for the detection of diabetic peripheral neuropathy. J Korean Orthop Assoc.

[30] Singh N, Armstrong DG, Lipsky BA (2005). Preventing foot ulcers in patients with diabetes. JAMA.

[31] England JD, Gronseth GS, Franklin G, Miller RG, Asbury AK, Carter GT, Cohen JA, Fisher MA, Howard JF, Kinsella LJ, Latov N, Lewis RA, Low PA, Sumner AJ, American Academy of Neurology; American Association of Electrodiagnostic Medicine; American Academy of Physical Medicine and Rehabilitation (2005). Distal symmetric polyneuropathy: a definition for clinical research: report of the American Academy of Neurology, the American Association of Electrodiagnostic Medicine, and the American Academy of Physical Medicine and Rehabilitation. Neurology.

[32] Nahm FS (2022). Receiver operating characteristic curve: overview and practical use for clinicians. Korean J Anesthesiol.

[33] Makkar B, Kumar V, Saboo B, Agarwal S. RSSDI Clinical Practice Recommendations for the Management of Type 2 Diabetes Mellitus 2022. Int J Diabetes Dev Ctries. 2022;42(Suppl 1):1–143. 10.1007/s13410-022-01129-5.

[34] Abbott CA, Vileikyte L, Williamson S, Carrington AL, Boulton AJ (1998). Multicenter study of the incidence of and predictive risk factors for diabetic neuropathic foot ulceration. Diabetes Care.

[35] Armstrong DG, Lavery LA, Vela SA, Quebedeaux TL, Fleischli JG (1998). Choosing a practical screening instrument to identify patients at risk for diabetic foot ulceration. Arch Intern Med.

[36] Ghosal S, Stephens J, Mukherjee A (2012). Quantitative vibration perception threshold in assessing diabetic neuropathy: is the cut-off value lower for Indian subjects? [Q-VADIS Study].. Diabetes Metab Syndr.

[37] Crawford F, Cezard G, Chappell FM, Murray GD, Price JF, Sheikh A, Simpson CR, Stansby GP, Young MJ (2015). A systematic review and individual patient data meta-analysis of prognostic factors for foot ulceration in people with diabetes: the international research collaboration for the prediction of diabetic foot ulcerations (PODUS). Health Technol Assess.

[38] Feng Y, Schlösser FJ, Sumpio BE (2011). The Semmes Weinstein monofilament examination is a significant predictor of the risk of foot ulceration and amputation in patients with diabetes mellitus. J Vasc Surg.

[39] Pourhamidi K, Dahlin LB, Englund E, Rolandsson O (2014). Evaluation of clinical tools and their diagnostic use in distal symmetric polyneuropathy. Prim Care Diabetes.

[40] Wang F, Zhang J, Yu J, Liu S, Zhang R, Ma X, Yang Y, Wang P (2017). Diagnostic accuracy of monofilament tests for detecting diabetic peripheral neuropathy: a systematic review and meta-analysis. J Diabetes Res.

[41] Suda M, Kawakami M, Okuyama K, Ishii R, Oshima O, Hijikata N, Nakamura T, Oka A, Kondo K, Liu M (2021). Validity and reliability of the semmes-weinstein monofilament test and the thumb localizing test in patients with stroke. Front Neurol.

[42] Wang A, Lv G, Cheng X, Ma X, Wang W, Gui J, Hu J, Lu M, Chu G, Chen J, Zhang H, Jiang Y, Chen Y, Yang W, Jiang L, Geng H, Zheng R, Li Y, Feng W, Johnson B, Wang W, Zhu D, Hu Y (2020). Guidelines on multidisciplinary approaches for the prevention and management of diabetic foot disease. Burns Trauma.

[43] Burgess J, Frank B, Marshall A, Khalil RS, Ponirakis G, Petropoulos IN, Cuthbertson DJ, Malik RA, Alam U (2021). Early detection of diabetic peripheral neuropathy: a focus on small nerve fibres. Diagnostics.

[44] Gin H, Rigalleau V, Baillet L, Rabemanantsoa C (2002). Comparison between monofilament, tuning fork and vibration perception tests for screening patients at risk of foot complication. Diabetes Metab.

[45] Richard JL, Reilhes L, Buvry S, Goletto M, Faillie JL (2014). Screening patients at risk for diabetic foot ulceration: a comparison between measurement of vibration perception threshold and 10-g monofilament test. Int Wound J.

[46] International Working Group on the Diabetic Foot. IWGDF guidelines on the prevention and management of diabetic foot disease. Accessed 10 Jan 2023. https://iwgdfguidelines.org/wp-content/uploads/2019/05/IWGDF-Guidelines-2019.pdf.

[47] Ishibashi Fukashi, Taniguchi Miki, Kosaka Aiko, Uetake Harumi (2019). Mitra Tavakoli; improvement in neuropathy outcomes with normalizing HbA1c in patients with type 2 diabetes. Diabetes Care.

[48] Dahlin LB, Elgzyri T, Löndahl M, Ekman L, Lindholm E (2020). Improved metabolic control using glucose monitoring systems leads to improvement in vibration perception thresholds in type 1 diabetes patients. Acta Diabetol.

[49] Fujita Y, Fukushima M, Suzuki H, Taniguchi A, Nakai Y, Kuroe A, Yasuda K, Hosokawa M, Yamada Y, Inagaki N, Seino Y (2008). Short-term intensive glycemic control improves vibratory sensation in type 2 diabetes. Diabetes Res Clin Pract.

[50] Solanki JD, Doshi RD, Virani NR, Sheth NS, Dhamecha JK, Shah CJ (2022). Prevalence and correlates of vibration perception threshold based diabetic peripheral neuropathy in Gujarati urban population: a cross sectional study. J Family Med Prim Care.

[51] Ashok S, Ramu M, Deepa R, Mohan V (2002). Prevalence of neuropathy in type 2 diabetic patients attending a diabetes centre in South India. J Assoc Physicians India.

[52] Mohan G, Chandey M, Monga A, Dev P (2018). Comparative study of detection of diabetic neuropathy by clinical and nerve conduction study in type 2 diabetes mellitus patients. Int J Adv Med.

[53] Adgaonkar A, Dawange A, Adgaonkar S, Kale V, Shekokar P (2014). Clinical profile of peripheral neuropathy in diabetes mellitus by nerve conduction study. Sch J App Med Sci.

[54] Pradeepa R, Rema M, Vignesh J, Deepa M, Deepa R, Mohan V (2008). Prevalence and risk factors for diabetic neuropathy in an urban south Indian population: the Chennai Urban Rural Epidemiology Study (CURES-55). Diabet Med.

[55] Srinivasan S, Raman R, Kulothungan V, Pal SS, Roy R, Ganesan S, Sharma T (2019). Four-year incident neuropathy and its risk factors in subjects with type 2 diabetes. J Assoc Physicians India.

