# Supplementary figures and images for: Assessment of the diagnostic accuracy of Vibrasense compared to a biothesiometer and nerve conduction study for screening diabetic peripheral neuropathy
Source: J Foot Ankle Res. 2023 Sep 28;16:65. doi: 10.1186/s13047-023-00667-3 (PMC10537102; doi:10.1186/s13047-023-00667-3)

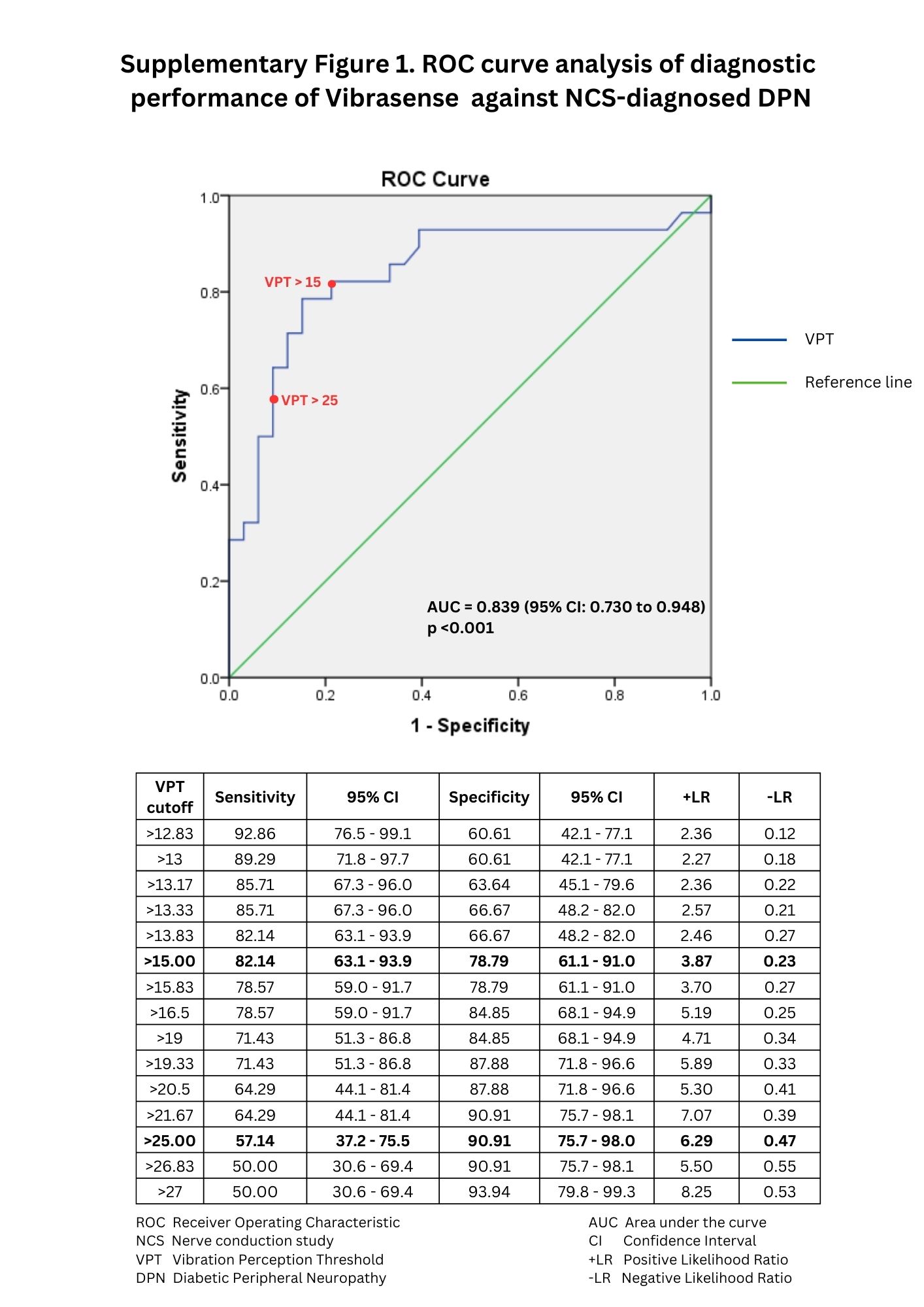

Supplement: Supplementary file 1 — Additional file 1. ROC curve analysis of diagnostic performance of Vibrasense against NCS-diagnosed DPN. ROC curve analysis was used to evaluate the diagnostic performance of Vibrasense against NCS-diagnosed DPN. The green diagonal line represents a classifier that makes random predictions. The blue line illustrates the diagnostic performance at different VPT levels of Vibrasense. The closer this curve is to the upper left corner of the plot, the better the performance. [file 13047_2023_667_MOESM1_ESM.jpg]
